# Supplementary material for: Yiai Fuzheng decoction inhibits triple-negative breast cancer by remodeling the immune microenvironment
Source: Front Immunol. 2025 Sep 30;16:1615631. doi: 10.3389/fimmu.2025.1615631 (PMC12518410; doi:10.3389/fimmu.2025.1615631)
Supplement: Supplementary file 2 [file DataSheet1.zip › Supplementary File 2/Treatment/C_vs_D/07_Pathway_Analysis/Enrichment_Analysis/predicted/Network.pdf]

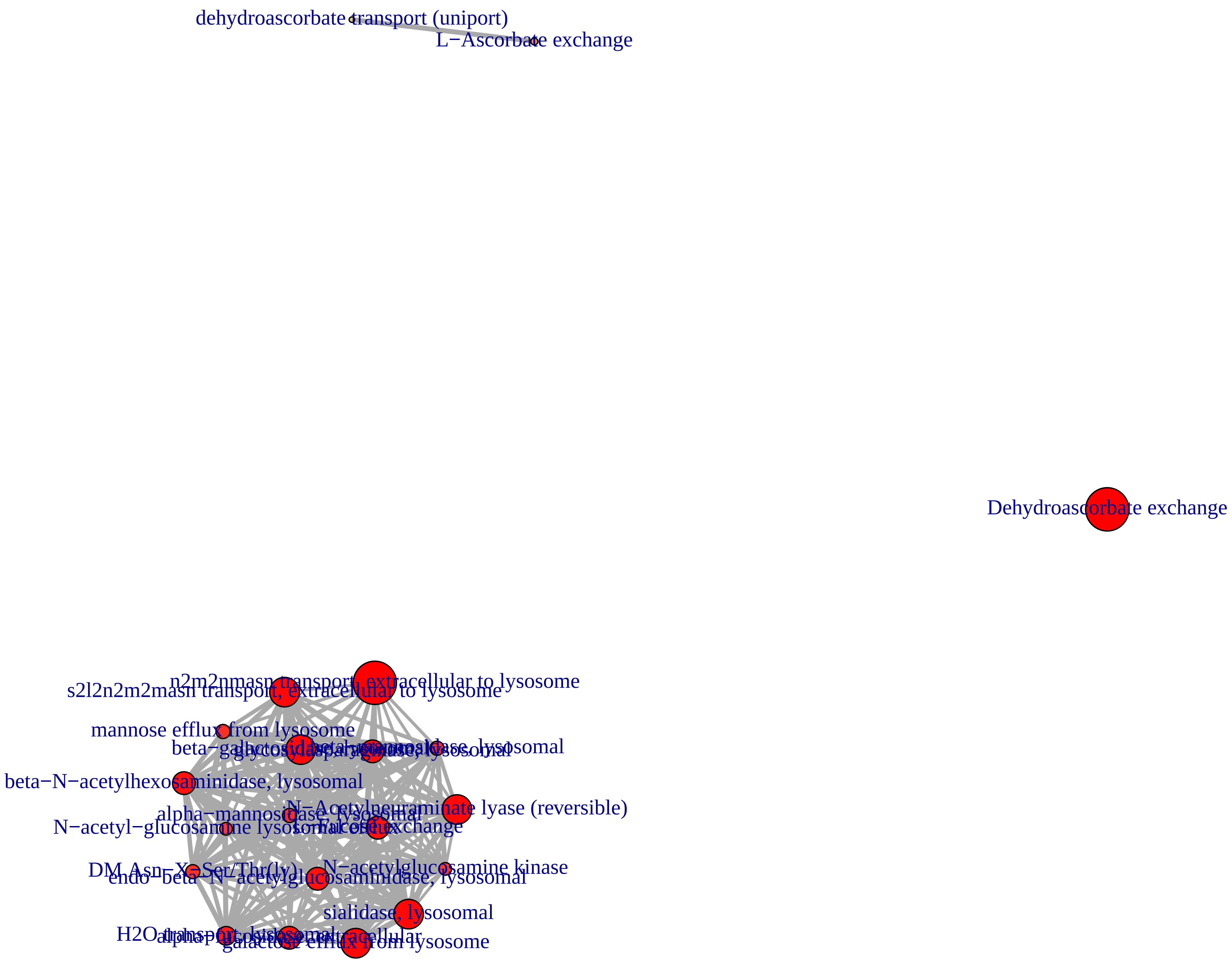
dehydroascorbate transport (uniport)
L−Ascorbate exchange
Dehydroascorbate exchange
n2m2nmasn transport, extracellular to lysosome
s2l2n2m2masn transport, extracellular to lysosome
mannose efflux from lysosome
glycosylasparaginase, lysosomal
beta−N−acetylhexosaminidase, lysosomal
N−Acetylneuraminate lyase (reversible)
alpha−mannosidase, lysosomal
N−acetyl−glucosamine lysosomal efflux
L−Fucose exchange
N−acetylglucosamine kinase
DM Asn−X−Ser/Thr(ly)
endo−beta−N−acetylglucosaminidase, lysosomal
sialidase, lysosomal
galactose efflux from lysosome
